# High-Grade Transformation in Adenoid Cystic Carcinoma of the Bartholin Gland: Case Report

**DOI:** 10.1055/s-0041-1736301

**Published:** 2021-12-21

**Authors:** Aline Evangelista Santiago, Nicky Teunissen, Bernardo Ferreira de Paula Ricardo, Eduardo Batista Cândido, Rafaela de Souza Furtado, Agnaldo Lopes da Silva Filho

**Affiliations:** 1 Gynecology Department, Universidade Estadual Paulista “Júlio de Mesquita Filho”, Botucatu, SP, Brazil; 2 Gynecology Department, Univeridade Federal de Minas Gerais, Belo Horizonte, MG, Brazil; 3 Pathology Department, Hospital MaterDei, Belo Horizonte, MG, Brazil; 4 Gynecology Department, Hospital MaterDei, Belo Horizonte, MG, Brazil

**Keywords:** adenoid cystic carcinoma, Bartholin gland, gynecology, oncology, vulvar neoplasms, carcinoma adenoide cístico, glândula de Bartholin, ginecologia, oncologia, neoplasias vulvares

## Abstract

**Introduction** In the present study, we report a case of primary adenoid cystic carcinoma (ACC) of the Bartholin gland with high-grade transformation (HGT). Adenoid cystic carcinoma of the Bartholin gland is a rare tumor and HGT has only been reported in head and neck tumors.

**Case Report** A 77-year-old woman with a non-ulcerated vulvar lesion on the topography of the right Bartholin gland. The patient was submitted to tumor resection followed by V–Y island flap and adjuvant radiotherapy. The histopathological examination revealed primary ACC of the Bartholin gland, with areas of HGT and extensive perineural invasion. The immunohistochemical study with p53 showed a diffuse and strong positive reaction in areas with HGT. After 24 months of follow-up, the patient presented distant metastases and died, despite having undergone to chemotherapy.

**Conclusion** As far as we know, this case is the first description in the literature of HGT in ACC of the Bartholin gland, and HGT appears to be associated with tumor aggressiveness.

## Resumo

**Introdução** Este estudo relata o caso de um carcinoma adenoide cístico (CAC) de glândula de Bartholin com transformação de alto grau. O CAC de glândula de Bartholin é um tumor raro, e sua transformação de alto grau é relatada somente em tumores de cabeça e pescoço.

**Relato de caso** Paciente de 77 anos de idade, do sexo feminino, com lesão vulvar não ulcerada na topografia da glândula de Bartholin direita. A paciente foi submetida a ressecção do tumor e realização de retalho em V-Y, seguidas de radioterapia adjuvante. O exame histopatológico revelou CAC primário de glândula de Bartholin, com áreas de transformação de alto grau e invasão perineural. O estudo imunohistoquímico com p53 mostrou reação positiva difusa e intensa em áreas com transformação de alto grau. Após 24 meses de seguimento, a paciente apresentou metástases à distância e faleceu, apesar de ter sido submetida a quimioterapia.

**Conclusão** Pelo que sabemos, este caso é a primeira descrição na literatura de transformação de alto grau em CAC de glândula de Bartholin, e a transformação de alto grau parece estar associada à agressividade do tumor.

## Introduction

Primary adenoid cystic carcinoma (ACC) of the Bartholin gland is a rare tumor first described in 1859. 1 It corresponds to approximately 0.001% of all gynecological malignancies and to 2% to 7% of vulvar malignant tumors. Tumors of the Bartholin glands may have different histological forms, but approximately 80% consist of squamous-cell carcinomas and adenocarcinomas. 2 3 4 The diagnosis of Bartholin gland cancer is usually late, because it has a nonspecific clinical presentation and differential diagnosis with Bartholin gland cyst and Bartolinitis. As a consequence, it is often discovered at an advanced stage, with a risk of lymph node (inguinal and pelvic) or general (pulmonary and bone) metastases. 4

The present article is the report of a rare case of ACC with high-grade transformation (HGT-ACC), which has only been reported in carcinomas in other regions, such as the head and neck. To our knowledge, this is the first reported case of HGT-ACC of the Bartholin gland.

## Case Report

A 77-year-old woman, nulliparous, postmenopausal but not undergoing hormonal therapy, with a history of infertility and resection of an ovarian cyst in 1966. In October 2016, the patient presented a non-ulcerated vulvar lesion of 5 cm in diameter on the topography of the right Bartholin gland, with apparent deep-layer infiltration without associated inguinal lymph node enlargement. Magnetic resonance imaging (MRI) and positron emission tomography-computed tomography (PET-CT) were performed, which showed a tumor measuring 4.0 × 2.7 × 3.8 cm on the topography of the right Bartholin gland topography, involving the distal third of the right labia majora of the vulva, the lower third of the vagina, and the distal urethra, with vulvar uptake with a standardized uptake value (SUV) of 11.22, without metastatic disease, lymph node involvement, or invasion of the mesorectal fascia and pelvic floor musculature. A biopsy of the lesion revealed a high-grade undifferentiated carcinoma in a limited material with no recognizable morphology of ACC.

**Fig. 1.**
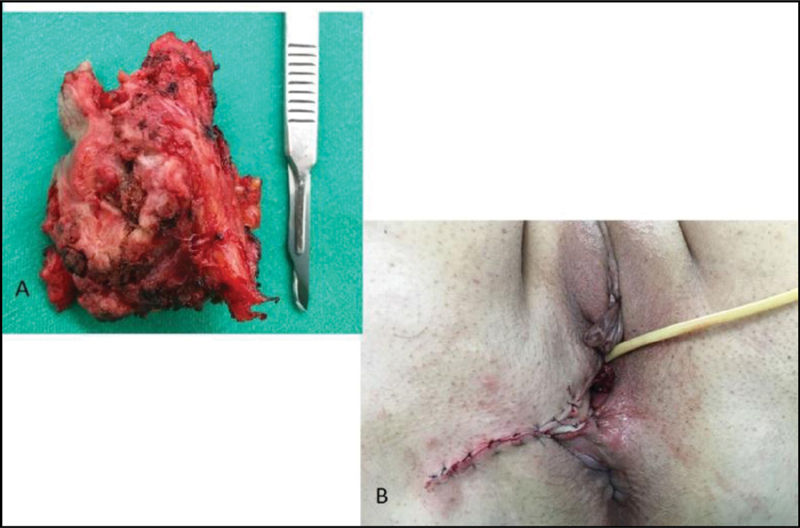
Surgical specimen ( **A** ) and immediate postoperative view of the reconstructed vulva with a V-Y island flap ( **B** ).

Tumor resection was performed under general anesthesia and spinal anesthesia. In the perioperative period, tumor invasion of the right ischioanal fossa was evidenced, with involvement of the levator ani muscle, extending up to the bladder, but without bladder invasion. Extended local excision of the tumor was performed with macroscopically free margins, without lymphadenectomy. At the end of the procedure, no gross macroscopic lesions were observed ( Fig. 1 ). The intraoperative analysis of a frozen section showed tumor-free surgical margins. We used the V-Y advancement flap from the medial thigh for vulvar reconstruction. Upon macroscopic inspection of the resected specimen, we established that the lesion was a solid mass measuring 5.0 × 4.0 × 3.0 cm, showing ulcerated tumor lesion measuring 4.0 × 3.5 cm, and distant 0.2 cm from the lowest margin.

**Fig. 2.**
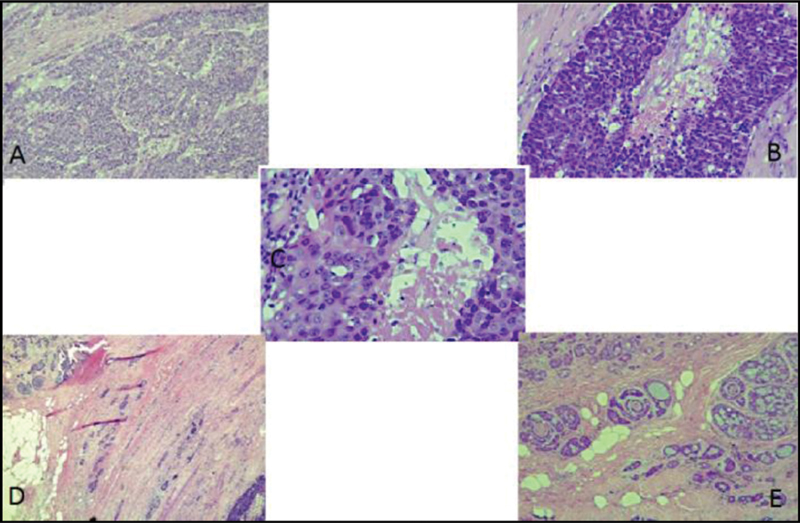
Microscopic features of the case herein reported. Histologic evaluation of primary adenoid cystic carcinoma of the Bartholin gland, with areas of high-grade morphology and extensive perineural invasion (hematoxylin-eosin, magnification ×100). ( **A** ) Area with well-differentiated tumor component; ( **B** , **C** , **D** ) areas with high-grade transformation; ( **E** ) transition area composed of well-differentiated components with high-grade transformation.

**Fig. 3.**
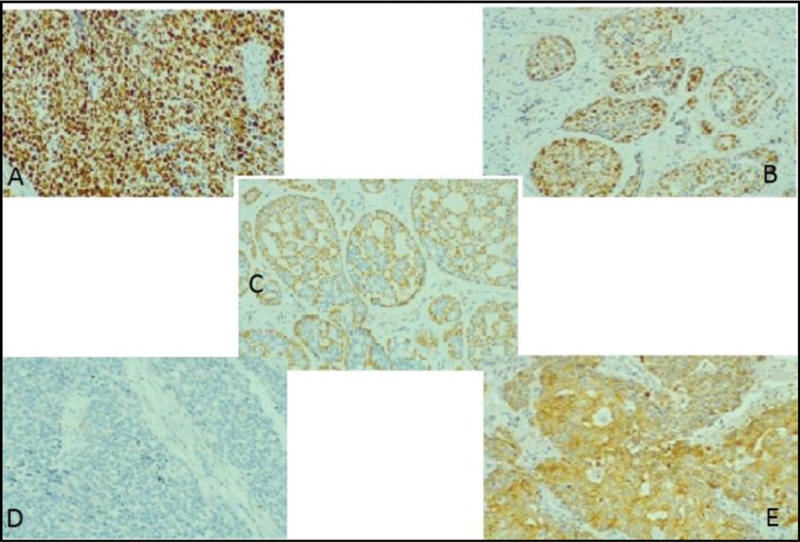
Immunohistochemical evaluation of primary adenoid cystic carcinoma of the Bartholin gland, with areas of high-grade morphology (magnification ×100). ( **A** ) Difuse positivity of p53; ( **B** ) p53 positivity in the well-differentiated component; ( **C** ) Immunostaining for p63 in the well-differentiated component; ( **D** ) p63 in the high-grade component; ( **E** ) pan cytokeratin AE1/AE.

The microscopic analysis of the histopathological examination revealed a primary ACC of the Bartholin gland, with areas of high-grade morphology, extensive perineural invasion, and presence of angiolymphatic emboli ( Fig. 2 ). The areas with conventional ACC had a morphological aspect ranging from tubular and cribriform areas to solid areas, and some transition areas composed of well-differentiated components with HGT were observed ( Fig. 2E ). The high-grade components were predominantly composed of solid architecture (71%) and highly atypical pleomorphic nuclei with a high number of mitoses. The immunohistochemical study with p53 showed a diffuse and strong positive reaction in HGT areas, and some tubular and cribriform areas also stained positively for p53 ( Fig. 3 ). In both the well-differentiated component and the HGT-ACC, P63 positivity was observed. The deep and circumferential margins of the surgical piece had no signs of neoplasia.

The patient received adjuvant treatment with external pelvic radiotherapy. Intensity-modulated radiation therapy (IMRT) was indicated and the dose was 45 Gy divided into 25 fractions of 1.8 Gy over the right inguinal region and the tumor bed. In addition, another boost of 14.4Gy was performed, divided into 8 fractions of 1.8Gy, reaching a total dose of 59.4Gy in the tumor bed, including the urethra. The patient presented good tolerance to the treatment, evolving only to dermatitis and moderate dysuria (according to the scale of the Radiation Therapy Oncology Group [RTOG]), and remained in clinical follow-up for 24 months, with no signs of relapse.

After the follow-up period, the patient remained asymptomatic, but presented signs of recurrence of the disease on PET-CT, with the presence of surgical-site hypermetabolism (SUV: 3.81) and bilateral pulmonary nodules (SUV: 3.35). Cisplatin and 5-fluorouracil (5-FU) chemotherapy was initiated. However, the patient died during treatment due to disease progression. Death occurred 36 months after the diagnosis.

## Discussion

Slow growth, high rate of local recurrence, perineural invasion, and usual late onset of distant metastases are known characteristics of ACC. 5 6 It is found mainly in the glandular tissues of the head and neck, especially in the salivary glands. However, in other tissues, such as in the breast and uterine cervix, ACC can also be detected, and displays the same behavior as salivary gland carcinoma. 5 7 Histologically, ACCs are characterized by three different growth patterns: tubular, cribriform, and solid growth. The number of solid segments seems to be the most important prognostic factor for adverse events. 8 A study 9 on ACC in the salivary glands showed that distant metastases occurred in 73% of the patients with a solid growth pattern, compared to 17% of cases with tubular growth pattern, and 8% with cribriform growth pattern. In the case herein reported, we observed percentages of distribution of growth patterns similar to those reported in the literature, with a predominance of the solid pattern.

A rare phenomenon not reported in the traditional ACC classification is the undifferentiation or HGT. The first description of this phenomenon in a case of ACC was made in 1999. 10 High-grade transformation is defined as the abrupt change of a well-differentiated tumor into undifferentiated morphology that does not have the original histological characteristics, making high-grade morphological areas usually well demarcated. However, in some cases, it is possible to identify a transition zone for a more typical morphology with cribriform and tubular areas, with frequent perineural invasion. 8 Although not easily identified, some transition areas, composed of well-differentiated components with HGT, were observed in the microscopic analysis of the tumor of the case herein reported ( Fig. 2E ).

The histological features present in HGT areas are thickening or irregularity of the nuclear membranes, prominent central nucleoli, necrosis, and microcalcifications. In addition, some features are considered major criteria, such as increase in nuclear size, confluent solid nodules, incomplete and focally absent luminal cell layers, increased Ki-67 labeling, fibrocellular desmoplastic stroma, micropapillary, squamous areas, and overexpression of p53. 11 In the present case, although the electron microscopic findings of such tumors revealed that the nuclear membranes were not thickened, such findings appear on hematoxylin-eosin (H&E) staining.

With the use of immunohistochemistry, specific tumor characteristics can be observed to distinguish well-differentiated areas of ACC from HGT-ACC. The p63 tumor marker, a myoepithelial marker, is present in well-differentiated ACC and absent or focally absent in HGT- ACC. 11 12 In the case herein reported, p63 positivity was observed in both the well-differentiated component and the HGT-ACC. Another marker that can be used for this identification is Ki-67, most expressed in areas with HGT. 12 In addition to these, some studies have shown that p53 genes have increased expression in the high-grade component, although other studies show that this marker may also have increased expression in well-differentiated ACC. 8 10 11 12 There is evidence that overexpression of the p53 gene in well-differentiated ACC is accompanied by a worse prognosis. Thus, changes in the p53 gene may be associated with the appearance of undifferentiated tumor cells, characterizing a marker of poor prognosis. 13 14 In the case herein reported, a diffuse positivity was observed for p53 in the well-differentiated component, which may have contributed to the poor outcome.

To differentiate between HGT-ACC and solid ACC, in addition to the immunohistochemical characteristics, some cellular characteristics can be evaluated. Nuclear increase, irregularities and necrosis of chromatin appear to be more pronounced in HGT-ACC. In addition, confluent sheets, fibrocellular desmoplastic stroma, necrosis, and high mitotic activity are also found in HGT-ACC. In cases in which the transition from well-differentiated ACC to HGT-ACC is not as abrupt as described in most cases, some characteristics can be found in both HGT-ACC and solid ACC, such as pleomorphism, mitotic activity, and focal necrosis. 12 In the present case, the microscopic analysis of the histopathological examination of tumor recurrence identified solid areas with pleomorphic areas with high mitotic index.

HGT-ACC is a variant of ACC with a more aggressive behavior, with a high probability for the development of recurrences and metastases. 11 15 A study 11 on subjects with head and neck HGT-ACC showed that approximately 57% of these patients developed lymph node metastases, compared to 5% to 25% of those with well-differentiated ACC. In addition, this study 11 indicated that 81% of the patients reported in the literature with HGT-ACC had recurrence of the disease. Of these 81%, 56.3% presented local recurrence, 57.9% presented lymph node metastasis, and 47.1% were diagnosed with distant metastases. 11 The most common site of metastasis is the lung. 2 The patient described in the present study presented both local recurrence and distant metastasis at the most common site.

Since ACC is a rare tumor and there are no prospective and randomized controlled trials on it, the treatment is not consensual, 4 but surgical resection is the preferred treatment. It can be performed with wide local excisions, hemivulvectomy, simple vulvectomy, and radical vulvectomy with and without inguinal and/or femoral lymphadenectomy. In patients with deep local infiltration, positive incisional margin or recurrence, adjuvant radiotherapy or chemotherapy can be performed. 2 3 The patient herein described underwent extended local excision of the tumor followed by adjuvant radiotherapy as the primary treatment, and chemotherapy after tumor recurrence. Considering the lack of consensus on treatment recommendations for Bartholin gland carcinomas and negative PET-CT for lymph node involvement, we chose not to perform lymphadenectomy. When we compare our case to a series of cases of Bartholin gland carcinomas described in the literature, 7 we observe coincident recurrence sites (the vulva and lung). However, the patient in the case herein presented was older when compared to the average age observed in the literature (72 years against 57 years), and the follow-up was shorter in our case (36 months against 41.9 months) than in in the case series mentioned. 7 This shorter follow-up was due to the patient's death.

## Conclusion

We reported a case of a 77-year-old woman with a primary ACC of the Bartholin gland, with areas of high-grade morphology, extensive perineural invasion, and presence of angiolymphatic emboli. To our knowledge, this is the first case of HGT reported in ACC of the Bartholin gland. Our patient was treated with extended local excision of the tumor, with free margins, followed by adjuvant radiotherapy. After 24 months of follow-up, the patient presented distant metastases, and the outcome was death, despite the chemotherapy received, reinforcing the aggressiveness of the tumor and probably of the HGT. A rare tumor, ACC of the Bartholin gland has no established diagnostic methods or defined treatment standards. Further research is needed to improve the understanding of the incidence and prognosis of HGT-ACC in gynecological tumors, including those of the Bartholin glands.
